# 

**Published:** 1875-04-01

**Authors:** 


*ORIGINAL COMMUNICATIONS:*

Extracts from an Inaugural Thesis on Yellow Fever. By W. H. H. Hutton, M.D. ........ 177

*EDITORIAL DEPARTMENT:*

The Way They Do It ........ 188

Commencement Exercises of the Chicago Medical College ........ 190

Ague as a Providential Dispensation ........ 191

*CORRESPONDENCE:*

A Letter from Paris ........ 192

*SOCIETY REPORTS:*

Transactions of the Chicago Society of Physicians and Surgeons. Regular Meeting, March 22, 1875 ........ 195

The Alumni Association of the Chicago Medical College ........ 203

*MISCELLANEOUS:*

American Medical Association ........ 204

*BOOK REVIEWS:* ........ 206

